# Declining incidence but little change in years lived with dementia in two German cohorts diagnosed with dementia in 2006/2008 and 2016/2018

**DOI:** 10.1186/s13195-025-01911-7

**Published:** 2025-12-02

**Authors:** Gabriele Doblhammer, Annette Erlangsen, Anne Fink, Vladimir Canudas-Romo

**Affiliations:** 1 Institute for Sociology and Demography, Rostock University, Ulmenstraße 69, Rostock, 18057 Germany; 2 German Center for Neurodegenerative Diseases, Venusberg-Campus 1/99, Bonn, 53127 Germany; 3 Psychiatric Center Copenhagen, Danish Research Institute for Suicide Prevention, Gentofte Hospitalsvej 15,4, Hellerup, DK-2900 Denmark; 4 School of Demography, Australian National University, 146 Ellery Cres, Canberra, Acton, ACT 2601 Australia

**Keywords:** Time trends, Dementia incidence, Dementia mortality, Life expectancy, Germany

## Abstract

**Background:**

We examined whether changes in dementia incidence and mortality have led to changes in the average number of years lived with and without dementia over a ten-year period in Germany.

**Methods:**

We calculated dementia rates using two samples of people aged 65 + from a German health insurance company, each comprising more than 100,000 people in 2006/08 and 2016/18. We examined time trends using negative binomial regression models and estimated average life expectancy (ALE) for people with and without dementia by fitting a three-stage Markov health-disease model.

**Results:**

Dementia incidence rates decreased by 7.3% in men and by 9.7% in women. Dementia mortality rates fell by 12% in men but did not change significantly in women. Non-dementia mortality rates fell by 7% in both men and women. The combined changes resulted in stable total ALE in men and increasing total ALE in women, whereas ALE with dementia increased in men and remained stable in women.

**Conclusions:**

The observed decline in dementia incidence did not translate into a reduction in the burden of the disease.

**Supplementary Information:**

The online version contains supplementary material available at 10.1186/s13195-025-01911-7.

## Background

Population ageing and continued gains in life expectancy have led to an overall increase in the prevalence of dementia among older adults. In Europe, approximately 10 million people are currently estimated to be living with dementia, while the number is expected to almost double by 2050 [1]. Due to its symptoms and high multimorbidity, dementia is one of the most care and cost intensive chronic diseases at old age, which poses a high burden on both informal as well as the general health care system [2].

Prediction models suggest that the increasing numbers of people with dementia is mainly driven by future trends in life expectancy [3], and that a substantial reduction in the prevalence of dementia is needed to offset this. Findings from meta-analyses reveal that the age-specific prevalence of dementia has either decreased or remained stable over the past two decades [4], whereas the age-specific dementia incidence rate decreased by approximately 13% per decade between 1988 and 2015 [4], albeit a slightly more accentuated decrease has been observed among women [5, 6]. However, the incidence of dementia increased by 25% in the UK between 2008 and 2016, following a decline of almost 29% between 2002 and 2008 [6].

Although recent trends suggest that dementia incidence is declining, it remains to be determined whether the absolute number of years lived with dementia is changing. To assess this, analyses of trends in age-specific dementia and non-dementia mortality are needed, and this is challenged by a lack of information. A decrease in dementia incidence would per se lead to an increase in years lived without dementia if non-dementia mortality declined or remained stable. It would also effectuate a decrease in years lived with dementia if trends in dementia mortality were stable or increasing. While people with dementia generally have higher mortality than people without dementia [2], trends over time are inconclusive. It is plausible that the mortality rate of people with dementia has declined in a similar manner as the one observed for people without dementia (for a review see [7–9]), but there are also reports of increases in mortality of women with dementia [10] and people with cognitive impairment [11]. Few studies have looked at the combined effect of dementia incidence and dementia mortality on life expectancy with dementia and found that it has decreased, particularly for women [10, 12], but there is also evidence of a small increase [8]. While evidence regarding life expectancy and potential years of life lost due to dementia exist, trends over time [13–15] of these measures remain to be assessed.

Above mentioned studies used prevalence-based measures to present the overall population-level burden of dementia, for instance by listing the total number of years lived with dementia or lost due to dementia in the population. However, we propose to use a more granular measure, average life expectancy, which provides insights into the individual-level burden and disease dynamics.

The aim of the study is therefore threefold: First, we examined whether changes in dementia incidence and mortality have led to changes in the average number of years lived with and without dementia in a German health insurance population. Second, we estimated the average number of years lived with dementia, which explicitly accounts for variation in age at disease onset and facilitates a direct comparison with average survival following a dementia diagnosis. Third, we examined whether these changes may have resulted in a change in the excess life years lost by those with dementia when compared with those without dementia, in absolute and relative terms. Germany has the fastest ageing population in Western Europe in terms of the old age dependency ratio of people aged 65 and over to those aged 20–64 [16] and is, therefore, optimally suited for this analyses.

## Methods

### Data

The scientific research institute “Wissenschaftliches Institut der Ortskrankenkassen” (WIDO) of the largest health insurance company in Germany, drew two random samples of 250,000 persons aged 50 and above for 2004 and 2014 and followed them until 2009 and 2019, respectively. The data included information on sex, date of birth and date of death by the exact month, and medical diagnoses based on the “International Classification of Disease, German Modification [17] on a quarterly basis.

#### Ethics Statement

This is an observational study which involved retrospective, anonymized claims data. It fell outside the scope of the Declaration of Helsinki and did not require ethical review.

Access to the data was legally approved by the WIDO (granted on 4 February 2021). The study was based on administrative claims data in which patients were never directly involved and data were fully anonymized before analyses. Individual patients cannot be identified during or after data collection, and the analyses presented do not affect patients whose anonymized records were used. Participant consent was not required. The University of Rostock Research Ethics Committee confirmed that no ethical approval is required.

### Analysis sample

Starting in 2004 and 2014, we implemented a two-year wash-out period to identify individuals aged 65 and over without dementia in the beginning of the baseline years 2006 and 2016. Also, the last years 2009 and 2019 were only used for validation purposes (see below in 2.3). This implied that for analysis 123,637 individuals were included from the first random sample who were observed from 2006 until 2008. From the second random sample, we followed 116,196 individuals from 2016 until 2018 (Table 1). A total of 705,109 person-years at risk of dementia or death were observed in the two samples. We identified 17,994 incidents of dementia with 22,688 person-years at risk of death, of which 5,433 died. Among people with no dementia, 24,301 deaths were observed.

**Table 1:** Person-years at risk and number of cases of incident dementia (DI), death with (DM) and without dementia (non-DM) and respective rates per 1,000 person-years

	Men		Women		Total
	Period 1	Period 2	Period 1	Period 2	Period1 & Period2
Dementia Incidence (DI)					
Person-years at risk	145,876	139,115	224,367	195,751	705,109
Cases	2,823	3,236	6,144	5,791	17,994
Incidence	19.4	23.3	27.4	29.6	25.5
95% CI Incidence	18.6–20.1	22.5–24.1	26.7–28.1	28.8–30.4	25.1–25.9
Dementia Mortality (DM)					
Exposures	3,407	3,956	8,028	7,297	22,688
Cases	974	1,077	1,698	1,684	5,433
Death Rate	285.9	272.2	211.5	230.8	239.5
95% CI Death Rate	267.9–303.8	256.0–288.5	201.5–221.6	219.8–241.8	233.1–245.8
Non-Dementia Mortality(non-DM)					
Exposures	145,876	139,115	224,367	195,751	705,109
Cases	5,806	6,023	6,455	6,017	24,301
Death Rate	39.8	43.3	28.8	30.7	34.5
95% CI Death Rate	38.8–40.8	42.2–44.4	28.1–29.5	30.0–31.5	34.0–34.9

*CI* Confidence interval

### Dementia

Dementia was identified using the following ICD-10 codes: G30, F00, F01, F02, F03, F05.1. Based on a previously documented validation strategy [10, 18], only internally validated diagnoses of dementia were included. Operationally, this implied that only diagnoses marked as “verified” by a medical doctor were included from outpatient services. For patients seen in inpatient services, only discharge and secondary diagnoses were considered. Also, only diagnoses, which had been recorded two times during the same quarter, i.e. either by different physicians or at separate occasions, or during the observation period were included. The sole exception to this criterion was for patients who had been recorded with one dementia diagnosis and passed away during the same quarter.

### Mortality follow-up

Information on deaths was given by the exact month which allowed us to calculate age for one-year age groups based on the date of birth, the date of death, the date of the end of the observation period or change of the health insurer, whichever was first.

### Analysis strategy and methods

Our analysis strategy was threefold: First, we calculated sex- and age-specific rates; second, we explored time trends using a negative binomial regression model; and third, we estimated average life expectancy for people with and without dementia using a three-stage Markov health-disease model.

#### Incidence and death rates

To estimate sex- and age-specific dementia incidence rates, denoted DI(x), numbers of men and women with a first valid diagnosis of dementia at age x, were divided by the respective person-years at risk at that age. To estimate sex- and age-specific death rates for those with incident dementia, denoted DM(x), the numbers of deaths at age x for men and women with dementia, were divided by the person-years at risk of dying for those with dementia at that age. We followed a similar approach for the death rate of those without dementia, denoted non-DM(x).

#### Negative binomial regression models

To identify time trends in DI(x), DM(x), and non-DM(x), we estimated sex-specific rate ratios (RR) from separate negative binomial regression models which included age and period as covariates. Age was defined as a second-degree polynomial centred at the mean age of the population exposed. For non-DM(x) and DI(x) this was 73.92 years for men and 75.87 years for women, for DM(x) it was 79.67 years for men and 82.99 years for women. Period was defined as a dummy variable taking the value of one for the years 2016–2018.

#### Average life expectancy (ALE)

To obtain the ALE for individuals with and without dementia, we fitted a three-stage Markov health-disease model with transition rates for: (1) non-dementia to dementia (dementia incidence, DI(x)), (2) dementia to death, DM(x), and (3) non-dementia to death, non-DM(x), between ages 65 and 100. In preliminary analyses, it was identified that rates at ages 80 and over had random fluctuations and smoothing was applied between ages 80 and 100. Smoothing was conducted using the Kannisto logistic model of mortality, which is a standard model for life table analysis [19]. Let $\:{{}_{35}\text{e}}_{65}$ denote life expectancy between ages 65 and 100 for those diagnosed with dementia, where 35 corresponds to the number of years between the two ages. Similar life expectancies can be calculated for all ages between 65 and 100, denoted as $\:{{}_{(100-\text{x})}\text{e}}_{\text{x}}$ for age x, which then are combined into an ALE for dementia. This ALE is weighted by the number of new incidents of dementia at each age x from the health-disease model, or decrements from the state of non-dementia to dementia at age x, denoted$\:\:{\text{d}}_{\text{x}},\:$as

1 $$\:{{}_{35}\stackrel{-}{\text{e}}}_{65}=\sum\:_{\text{x}=65}^{100}{{}_{(100-\text{x})}\text{e}}_{\text{x}}\:{\text{d}}_{\text{x}.}$$

This implies that the life expectancy between ages 65 and 100, or $\:{{}_{35}\text{e}}_{65}$, is weighted by the number of new dementia cases in the health-disease model at that age, $\:{\text{d}}_{65}$, added to this is the life expectancy between ages 66 and 100, which is weighted by the number of new dementia cases in the health-disease model at age 66, as $\:{{}_{34}\text{e}}_{66}\:{\text{d}}_{66}$, and so on until age 100. This approach returns an ALE with dementia that takes into account the age at diagnosis of individuals. Opposing this would be to take life expectancy at age 65, which assumes that all individuals were diagnosed dementia at that age.

Let now $\:{{}_{(100-\text{x})}\text{e}}_{\text{x}}^{\text{*}}$ denote life expectancy between ages x and 100 for the population without dementia. An ALE is then calculated as $\:{{}_{35}\stackrel{-}{\text{e}}}_{65}^{\text{*}}$ where the weights are the same as in Eq. (1) for the dementia population. The difference between the ALE for the populations with and without dementia returns our measure of excess life years lost, $\:\text{L}\text{Y}\text{L}={{}_{35}\stackrel{-}{\text{e}}}_{65}^{\text{*}}-{{{}_{35}\stackrel{-}{\text{e}}}_{65}}_{}^{}$. Efforts of calculating the ALE and excess life years lost have been presented before [20, 21]. In this study, we improved the methodology by using decrements in the health-disease model to eliminate the confounding effect of the structure of the population from using observed dementia incidence cases.

In addition to the excess life years lost, we calculated the ratio of ALE for those with dementia over the total number of years lived by the study population, or $\:\text{R}=\frac{{{}_{35}\stackrel{-}{\text{e}}}_{65}^{}}{{{}_{35}\stackrel{-}{\text{e}}}_{65}^{\text{*}}+{{}_{35}\stackrel{-}{\text{e}}}_{65}^{}}$, representing the proportions of years lived with dementia. To obtain confidence intervals, we bootstrapped the age-specific number of incident dementia cases and dementia deaths assuming a binomial distribution with 1000 replications. Significance was assessed in terms of non-overlap of confidence intervals. All calculations were performed in R 4.3.2 and Stata 17. 0.

## Results

### Descriptives

#### Dementia incidence (DI)

In the period 2006/08, 145,876 person-years of men were at risk of receiving a diagnosis of incident dementia with a decline to 139,115 in 2016/18. Among women, the at-risk population decreased from 224,367 to 195,751 person-years (Table 1). The number of incident dementia cases increased from 2,823 to 3,236 for men and decreased from 6,144 to 5,791 for women. Dementia incidence rates at age 65 years and over increased from 19.4 (95% CI, 18.6–20.1) to 23.3 (95% CI, 22.5–24.1) per 1,000 person-years lived for men and increased from 27.4 (95% CI, 26.7–28.1) to 29.6 (95% CI, 28.8–30.3) for women.

#### Dementia mortality (DM)

In the first period, there were 974 deaths out of 3,407 person-years in men with incident dementia. During the second period, 1,077 of the 3,956 male person-years at risk died. Among the 8,028 female person-years with incident dementia in the first period, 1,698 died, while 1,684 out of 7,297 person-years died in the second period. Thus, the dementia mortality rate declined from 285.9 (95% CI, 267.9–303.8) to 272.2 (95% CI, 256.0–288.5) per 1,000 person-years for men, and increased from 211.5 (95% CI, 201.4–221.6) to 230.8 (95% CI, 219.8–241.8) for women.

#### Non-dementia mortality (non-DM)

In the non-dementia population 5,806 men and 6,455 women died during the first period, while 6,023 men and 6,017 women died during the second period; implying that death rates increased from 39.8 (95% CI, 38.8–40.8) to 43.3 (95% CI, 42.2–44.4) per 1,000 person-years for men, and from 28.8 (95% CI, 28.1–29.5) to 30.7 (95% CI, 30.3–31.5) for women. Note that the at-risk person-years for this segment corresponds to those listed above for being at risk of incident dementia.

### Rate ratios (RR) of time trends in dementia incidence, dementia mortality, and non-dementia mortality

Over the ten-year period (Table 2), the age-adjusted incidence rate for dementia decreased significantly by 7.3% for men (RR = 0.927; 95% CI, 0.887–0.970; p-value < = 0.001) and by 9.7% for women (RR = 0.903; 95% CI, 0.859–0.950; p-value < = 0.001). At the same time, the dementia mortality rate decreased significantly by 12% for men (RR = 0.880; 95% CI, 0.809–0.957;p-value = 0.003) but did not change significantly for women (RR = 1.062; 95% CI, 0.980–1.151; p-value = 0.141). Non-dementia mortality decreased by 7% for both men (RR = 0.931; 95% CI, 0.894–0.971; p-value < = 0.001) and women (RR = 0.937; 95% CI, 0.886–0.991; p-value = 0.022). All time trends are clearly reflected in the age-specific rates of the two periods (Supplementary Material SFig. 1 for dementia incidence, SFig. 2 for dementia mortality, and SFig 3. for non-dementia mortality).

**Table 2:** Rate ratios (RR) and 95% confidence intervals of trends in dementia incidence (DI), dementia mortality (DM), and non-dementia mortality (non-DM) by sex from negative binomial regression models

	Men				Women			
	RR	LCI	UCI	*p-*value	RR	LCI	UCI	*p*-value
Dementia incidence (DI)								
Age	1.151	1.143	1.158	0.000	1.169	1.160	1.177	0.000
Age*Age	0.998	0.998	0.999	0.000	0.997	0.997	0.998	0.000
2016–2018 (Ref: 2006–2008)	0.927	0.887	0.970	0.001	0.903	0.859	0.950	0.000
Constant	0.017	0.016	0.017	0.000	0.021	0.020	0.021	0.000
Dementia mortality (DM)								
Age	1.054	1.047	1.061	0.000	1.072	1.064	1.079	0.000
Age*Age	1.001	1.000	1.001	0.039	1.001	1.000	1.002	0.036
2016–2018 (Ref: 2006–2008)	0.880	0.809	0.957	0.003	1.062	0.980	1.151	0.141
Constant	0.267	0.251	0.285	0.000	0.183	0.173	0.194	0.000
Non-Dementia mortality (non-DM)								
Age	1.085	1.082	1.088	0.000	1.113	1.109	1.117	0.000
Age*Age	1.001	1.001	1.001	0.000	1.001	1.001	1.001	0.000
2016–2018 (Ref: 2006–2008)	0.931	0.894	0.971	0.001	0.937	0.886	0.991	0.022
Constant	0.057	0.055	0.059	0.000	0.046	0.044	0.048	0.000

*LCI* 95% Lower confidence interval, *UCI* 95% Upper confidence interval

### Time trends in dementia life expectancy and non-dementia life expectancy

**Fig. 1:**
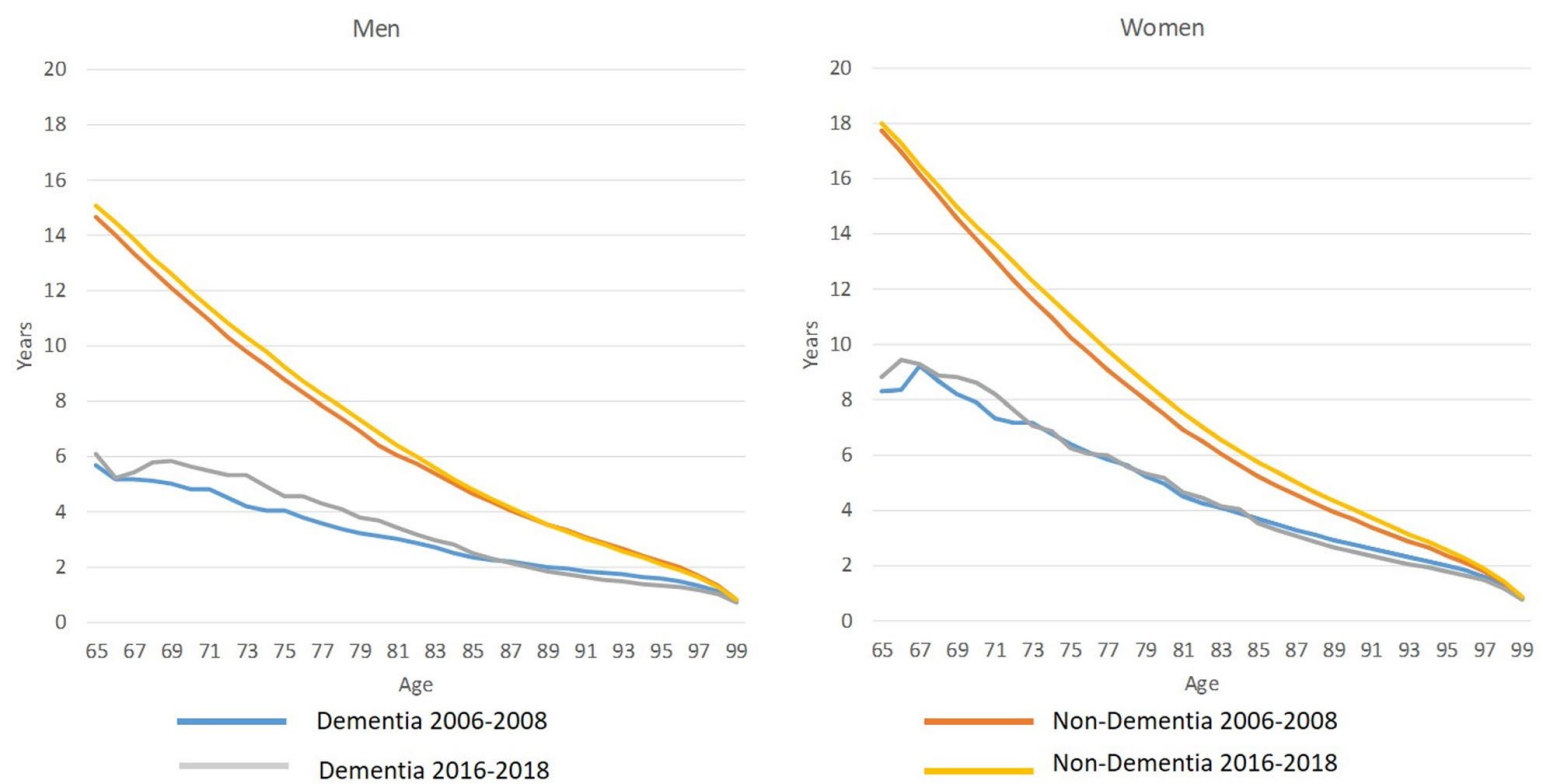
Dementia life expectancy ($\:{{}_{(100-\text{x})}\text{e}}_{\text{x}}^{}$) and non-dementia life expectancy ($\:{{}_{(100-\text{x})}\text{e}}_{\text{x}}^{\text{*}}$) for the periods 2006–2008 and 2026 − 2018 by sex

In the three-stage Markov health-disease model, life expectancy at age 65 (Fig. 1) increased from 5.67 years in period 1 to 6.08 years in period 2 for men with dementia and from 8.28 to 8.80 years for women with dementia. Life expectancy at age 65 of men without dementia increased from 14.63 to 15.08 years, while an increase from 17.75 to 18.00 years was observed for women. However, these were exact life expectancies for individuals at age 65. To obtain the average life expectancies, life expectancies at each age for individuals with and without dementia, or $\:{{}_{(100-\text{x})}\text{e}}_{\text{x}}^{}$ and $\:{{}_{(100-\text{x})}\text{e}}_{\text{x}}^{\text{*}}$ respectively, were weighted by the multi-state life table decrements.

### Time trends in average life expectancy (ALE)

Based on the multi-state life table, a statistically significant increase in ALE was observed. For the population of women, ALE increased from 11.19 (95% CI, 11.00–11.40) years in 2006–2008 to 11.70 (95% CI, 11.49–11.95) years in 2016-18. For men, it increased from 9.87 (95% CI, 9.67–10.12) years to 10.31 (95% CI, 10.08–10.57) years during the examined period (Table 3). Segregated by status of disease, ALE among women with dementia remained stable with 4.42 (95% CI, 4.27–4.59) years during the first period and 4.42 (95% CI, 4.27–4.62) years in the second. We found an increase in ALE for men with dementia; from 3.14 (95% CI, 3.02–3.31) years to 3.45 (95% CI, 3.30–3.63) years. Among individuals with no dementia, ALE increased from 6.76 (95% CI, 6.66–6.86) years to 7.28 (95% CI, 7.16–7.39) years among women and from 6.73 (95% CI, 6.58–6.88) years to 6.86 (95% CI, 6.72–7.00) years among men. These diverging trends were caused by sex-specific trends in dementia mortality, i.e. decreasing for males but stable for females. The combination of declining dementia incidence and mortality rates for men resulted in, on average, more years of life with dementia. For women, the decreasing dementia incidence coincided with stability in the dementia mortality rate, which led to a small decline in number of years lived with dementia albeit not statistically significant, and thus their ALE with dementia remained stable.

**Table 3:** Average life expectancy (ALE) and 95% confidence intervals of persons with and without dementia and of the total multi-state lifetable population

		Dementia			Non-dementia			Total		
Sex	Period	ALE	LCI	UCI	ALE	LCI	UCI	ALE	LCI	UCI
Men	2006–2008	3.14	3.01	3.30	6.73	6.58	6.88	9.87	9.67	10.12
Men	2016–2018	3.45	3.29	3.63	6.86	6.72	7.00	10.31	10.08	10.57
Women	2006–2008	4.42	4.27	4.59	6.76	6.66	6.86	11.19	11.00	11.40
Women	2016–2018	4.42	4.27	4.62	7.28	7.16	7.39	11.70	11.49	11.95

The differences between the ALE with and without dementia are the excess LYL of those with dementia (Table 4). For men excess LYL declined from 3.59 years (= 31.86–28.27) (95% CI,3.39–3.77) to 3.41 years (95% CI,3.22–3.58), however, the difference was statistically not significant. For women they increased statistically significant from 2.34 years (95% CI,2.16–2.49) to 2.86 years (95% CI,2.68–3.03).

**Table 4:** Excess life years lost (LYL) and ratio ALE dementia to ALE total

		Excess Life Years Lost			ALE Ratio		
Sex	Period	LYL	LCI	UCI	Dementia/Total	LCI	UCI
Men	2006–2008	3.59	3.39	3.77	0.32	0.31	0.33
Men	2016–2018	3.41	3.22	3.58	0.33	0.32	0.35
Women	2006–2008	2.34	2.16	2.49	0.40	0.39	0.40
Women	2016–2018	2.86	2.68	3.03	0.38	0.37	0.39

Regarding the ratio of ALE with dementia to total ALE, there were no significant changes between the two periods. The ratio increased for men from 0.32 (95% CI,0.31–0.33) to 0.33 (95% CI,0.32–0.35) and decreased for women from 0.40 (95% CI,0.39–0.40) to 0.38 (95% CI,0.37–0.39) years.

## Discussion

Using a sample from the largest German health insurance company, we found significant declines in the incidence of dementia for both sexes over a ten-year period from 2006/08 to 2016/18. This decline resulted in an increase in number of years lived without dementia. The decline did, however, not translate into a lower disease burden as measured by average years lived with dementia which increased among men and remained stable among women.

A previous study for Germany [10] found a declining trend in dementia incidence of 9% over a 3-year period from 2006/7 to 2009/10 for both men and women, which was larger than the decline reported in this study. However, declines of about 10% for women and 7% for men over a 10-year period reported here are comparable with findings from previous meta-analysis [5], which reported a pooled 10-year decline across five studies of 8% for women and 24% for men, and a combined decline of 14% for those aged 65 and over. One explanation for the lower decline in this study could be the introduction of a new billing code in Germany in 2013 [22], which provided an additional incentive for physicians to record dementia diagnoses. Another explanation may be that the decline slowed or even stopped during the second decade, as has been observed in the UK based on the English Longitudinal Study of Ageing [6].

The findings from our health-disease model emphasise that trends of dementia incidences do not convey complete information on the burden that dementia has on individuals and society; one has to also consider trends of dementia mortality. This fact has previously been under-recognised [11, 23, 24], possibly due to a lack of a comprehensive assessment, e.g. by not including all epidemiological measures [7, 9]. In the French Three-City Study sex-specific trends were observed: dementia mortality was found to decrease with 12% for men, which was statistically significant, but increased with 8% for women, which was not significant [8]. Previously and based on German data, a significant 20% increase in dementia mortality has been demonstrated for women, while an 11% increase for men did not render significant [10]. As billing codes have changed over time, with dementia receiving more attention in later years in Germany [22], dementia may now be diagnosed at an earlier stage than in the past. This would lead to a decrease in dementia mortality at a similar age. More research is needed to better understand the relationship between age at first diagnosis of dementia, disease severity and the resulting mortality.

By using average life expectancy instead of remaining life expectancy, we were able to account for the average number of years lived with dementia by accounting for onset of dementia at different ages. This offers a more precise estimate versus calculations based on a single decrement table where the number of years lived with dementia is likely to be over-estimated due to the assumption that onset of dementia occurred at a fixed age. The use of prevalence-based life expectancy (e.g [12])., would underestimate the average number of years lived with dementia because it gives the number of years lived with dementia in the total population, rather than the number of years lived with dementia among people with dementia. This is also the case for the multi-state approach used in a previous study based on health claims data [10] or in [25] based on the Rotterdam Study. However, the average lifespan can be compared with the average survival time after a dementia diagnosis which ranges from about 3 to 4 years in Canada [15], Sweden [13] and UK [26] to about 5/7 years in Norway [14] and the Rotterdam Study [27]. Our estimates of three to four years are well within this range.

In this sense, average life expectancy complements prevalence-based burden measures rather than replaces them. While prevalence informs the magnitude of burden of dementia at the population level, average life expectancy provides insight into the duration and experience of that burden among the individuals diagnosed with dementia. The latter provides central insights regarding the clinical and public health implications of the disease.

In our study, we use the difference between average life expectancy with and without dementia as a measure of life years lost due to dementia which we denote as excess LYL. We compare this measure to the measure of potential years of life lost (PYLL), based on a life table in which the observed number of deaths at a given age is multiplied by the remaining life expectancy at that age [28]: For Sweden [13] and the Netherlands [25] estimates are about 3.5 years lost due to dementia, which is close to our results (3.6 (period1)/3.4 (period2) years for men and 2.3 (period1)/2.9 (period2) years for women aged 65 and above). For Norway [14], estimated 9.8 years (95%CI: 9.1, 10.6) for men and 10.8 years (95%CI: 9.7, 11.9) for women at ages 70 and above.

Our trends in the ratio of average life expectancy with dementia to total average life expectancy support the dynamic equilibrium scenario of health development [29]. This scenario assumes that the ratio of years lived with dementia to total life years remains unchanged, with a shift from more severe to less severe morbidity. However, as people with dementia are now diagnosed later in life, this raises new questions about the severity of their dementia, their multimorbidity and their need for care. In Sweden, people aged 75 and over with incident dementia spent more than 2 years in moderate (14 months) and severe (12 months) stages of dementia [13]. Survival in moderate stages may become shorter as incidence shifts to older ages due to faster disease progression or generally higher mortality. In addition, dementia is associated with multimorbidity, which is increasing over time [30]. More research is needed to understand how the shift in dementia incidence to older ages relates to the different stages of dementia, the increase in multimorbidity, and whether the need for care has increased and/or the quality of life of people with dementia has deteriorated over time.

The present study has several strengths among them most importantly that health claims data cover the entire population, including residents of nursing homes, where the prevalence of dementia is significantly higher [31]. Using a random sample of the total population, we were able to reduce any sampling and selection bias and the large number of cases allowed detailed analysis of incidence and mortality in all age groups up to 100 years, with the oldest ages often under-represented in surveys. Limitations mainly result from the fact that health claims data are compiled for administrative purposes rather than disease documentation, implying that inaccuracies, including false positive and false negative diagnoses, cannot be excluded. Despite using an internal validation procedure to address false positive diagnoses [10], we were unable to compensate for the under-recordings of dementia diagnoses. Still, the prevalence and the incidence of dementia obtained from this data source were previously found to be on level with international studies [10, 32]. Our analyses did not account for change of health insurance, however, only 1.7% and 0.4% switched insurance company during the first and second period, respectively. While there were no significant legislative changes during the study period, a new billing code for geriatric care by primary care physicians was introduced in 2013 [22]. This led to a sudden spike in dementia diagnoses, which however, had attenuated by 2015, i.e. the onset of our second study period. Information on how these billing codes may have resulted in more and/or earlier diagnoses during 2013 and 2014 were not available. Still, it is possible that they may have biased our estimates in a conservative direction. Future simulation studies may help clarify the potential impact of these billing codes, not only on dementia but also on other related diagnoses. We did not perform sensitivity analyses, as any assumed trends would have been arbitrary and simulation studies would need a broader perspective, i.e. including other related diagnoses.

Another important drawback is the lack of information by etiology, with the large majority of the dementia diagnoses being unspecified dementia (ICD-10 F03). We also do not know the severity stage of dementia and mild cognitive impairment is not part of our study. In terms of representativity, the AOK population on average have lower socio-economic status than the general population and exclude civil servants and self-employed [33]. However, their mortality pattern is comparable to that of the general German population at ages 65 and above [34]. Our claims data do not contain information on the social status of the population, in particular on educational attainment. Given that larger declines in trends of dementia prevalence and incidence have been demonstrated for individuals with high educational level [6, 12], we may have underestimated the decline in dementia incidence in the German population.

Also the Markov health-disease model has three assumptions that need to be critically reflected: first, it assumes a stationary population with no changes in transition rates until the last member of the population has died, which is at odds with the trends we observe in dementia incidence and dementia mortality. Second, it uses a synthetic cohort from a cross-sectional perspective rather than following a cohort from age 65 to death. Third, it assumes that future states depend only on the current state, ignoring the duration in the current state. Finally, we only examined incident cases of dementia, i.e. all prevalent dementia cases were excluded. This was necessary because their date of onset was unknown. Prevalent dementia cases were also excluded from the calculation of dementia death rates because they were solely based on individuals who had been newly diagnosed with dementia within the last three years. The relationship between time since diagnosis of dementia and mortality is, however, complex because the mortality risk of people with dementia depends on a number of factors, including age at onset [35, 36], comorbidity [37] and disease severity [38, 39]. Therefore, more studies on the survival time after dementia diagnoses are needed to fully understand whether a declining incidence also leads to a reduced burden of dementia.

## Conclusions

Using German claims data, a declining incidence of dementia has been observed in recent years, leading to an increase in the absolute number of years individuals live without dementia. While this trend appears positive, the study highlights significant implications: the declining incidence has not resulted in a proportional reduction in the overall disease burden of dementia. Improvements in mortality rates mean that the number of years lived with dementia has remained relatively stable.

Moreover, by employing enhanced methodologies to assess changes in the disease burden over time, this study provides more precise estimates of life years lost due to dementia. Importantly, the findings reveal that the decline in incidence has not decreased the years of life lost to dementia. On the contrary, this burden has increased among women.

The persistence of this burden is influenced by complex factors, including mortality trends among individuals with dementia compared to those without, a dynamic that remains poorly understood. Additionally, the severity of dementia and associated multimorbidity—particularly as diagnoses occur at increasingly older ages—further shape the disease burden, an area where significant knowledge gaps exist.

In the absence of effective treatments for dementia, it is critical to deepen our understanding of protective factors that can delay dementia onset or contribute to its prevention. Addressing these gaps is essential for reducing the disease burden and improving outcomes for individuals and society.

## Supplementary Information

Supplementary Material 1

## Data Availability

The Scientific Research Institute of the AOK (WIdO) imposes strict rules on sharing health claims data, as these are classified according to ethical restrictions due to privacy concerns. Anonymized data are available to researchers and institutions upon request. In order to request access to the health claims data of the AOK, please contact the WIdO directly (http://www.wido.de/, mail: wido@wido.bv.aok.de).

## Abbreviations

ALE: Average life expectancy
CI: Confidence interval
DI: Dementia incidence
DM: Dementia mortality
LCI: Lower confidence interval
LYL: Life years lost
non-DM: Non–dementia mortality
PYLL: Potential years of life lost
RR: Rate ratio
UCI: Upper confidence interval
WIDO: Wissenschaftliches Institut der AOK
