# Supplementary material for: Declining incidence but little change in years lived with dementia in two German cohorts diagnosed with dementia in 2006/2008 and 2016/2018
Source: Alzheimers Res Ther. 2025 Dec 2;17:255. doi: 10.1186/s13195-025-01911-7 (PMC12670842; doi:10.1186/s13195-025-01911-7)
Supplement: Supplementary file 1 — Supplementary Material 1 [file 13195_2025_1911_MOESM1_ESM.docx]

SFig.1: Age-specific dementia incidence by sex: observed values and predictions from negative binomial regressions, AOK claims data

SFig.2: Age-specific dementia mortality rates by sex: observed values and predictions from negative binomial regressions, AOK claims data

SFig.3: Age-specific non-dementia mortality rates by sex: observed values and predictions from negative binomial regressions, AOK claims data
